# Using the Beta distribution in group-based trajectory models

**DOI:** 10.1186/s12874-018-0620-9

**Published:** 2018-11-26

**Authors:** Jonathan Elmer, Bobby L. Jones, Daniel S. Nagin

**Affiliations:** 1 Department of Emergency Medicine, Critical Care Medicine and Neurology, University of Pittsburgh, Pittsburgh, PA USA; 2 University of Pittsburgh Medical Center, Pittsburgh, PA USA; 3 Heinz College, Carnegie Mellon University, Pittsburgh, PA 15206 USA

**Keywords:** Group-based trajectory modeling, Beta distribution, Cardiac arrest

## Abstract

**Background:**

We demonstrate an application of Group-Based Trajectory Modeling (GBTM) based on the beta distribution. It is offered as an alternative to the normal distribution for modeling continuous longitudinal data that are poorly fit by the normal distribution even with censoring. The primary advantage of the beta distribution is the flexibility of the shape of the density function.

**Methods:**

GBTM is a specialized application of finite mixture modeling designed to identify clusters of individuals who follow similar trajectories. Like all finite mixture models, GBTM requires that the distribution of the data composing the mixture be specified. To our knowledge this is the first demonstration of the use of the beta distribution in GBTM. A case study of a beta-based GBTM analyzes data on the neurological activity of comatose cardiac arrest patients.

**Results:**

The case study shows that the summary measure of neurological activity, the suppression ratio, is not well fit by the normal distribution but due to the flexibility of the shape of the beta density function, the distribution of the suppression ratio by trajectory appears to be well matched by the estimated beta distribution by group.

**Conclusions:**

The addition of the beta distribution to the already available distributional alternatives in software for estimating GBTM is a valuable augmentation to extant distributional alternatives.

## Background

A trajectory describes the evolution of a behavior, biomarker, or some other repeated measure of interest over time. Group-based trajectory modeling (GBTM) [1], also called growth mixture modeling [2], is a specialized application of finite mixture modeling designed to identify clusters of individuals who follow similar trajectories. Originally developed to study the developmental course of criminal behavior [3], GBTM is now widely applied in biomedical research in such diverse application domains as chronic kidney disease progression [4], obesity [5, 6], pain [7], smoking [8], medication adoption and adherence [9, 10], and concussion symptoms [11].

Like all finite mixture models, GBTM requires that the distribution of the data composing the mixture be specified, although there are no theoretical limits on the distributions that could be used. In GBTM**,** parameters of the specified distribution (e.g. mean and variance of a normal distribution) are allowed to vary across trajectory groups. To our knowledge, previously published applications have all specified the normal distribution, perhaps with censoring, the Poisson distribution, perhaps with zero-inflation, or the binary logit function. Real-world continuous biomedical data are frequently not normally distributed even after allowing censoring. This is particularly true of biomarker data, which are generally positive, right skewed, and often zero-inflated. This creates a need for flexible alternatives to the Gaussian distribution [12].

In this article, we demonstrate an application of GBTM based on the beta distribution. It is offered as an alternative to the normal distribution for modeling continuous longitudinal data are poorly fit by other distributions. The primary advantage of the beta distribution is the flexibility of the shape of the density function. The normal density function, even in its censored form, must follow some portion of it familiar bell-shaped form whereas the shape of beta distribution is far less constrained. The disadvantage of beta distribution is that the data under study must be transformable to a 0–1 scale.

## Methods

The beta distribution can be parameterized in several different ways. One which is particularly useful for our purposes was proposed by [12]. Let y denote a beta distributed random variable:

$$ P\left(y;\mu, \phi \right)=\frac{\Gamma \left(\phi \right)}{\Gamma \left(\mu \phi \right)\Gamma \left(\left(1-\mu \right)\phi \right)}{y}^{\mu \phi -1} $$

where 0 < y < 1, 0 < μ < 1 and ϕ > 0. Under this parameterization E(y) = μ and *Var*(*y*) = *μ*(1 − *μ*)/1 + *ϕ*). The parameter ϕ is known as the precision parameter, because for any μ a larger value of ϕ results in a smaller Var(y).

We turn now to incorporating the beta distribution into GBTM. In describing a GBTM, we denote the distribution of trajectories by *P*(*Y*_*i*_), where the random vector *Y*_*i*=_(*y*_*i*1_, *y*_*i*1_, …*y*_*iT*_) represents individual *i*’s longitudinal sequence of measurements over T measurement occasions. The GBTM assumes that the population distribution of trajectories arises from a finite mixture composed of *J* groups. The likelihood for each individual *i*, conditional on the number of groups *J*, may be written as:

1 $$ P\left({Y}_i\right)=\sum \limits_{j=1}^J{\pi}_j\bullet P\left({Y}_i|j;{\theta}_j\right) $$

where *π*_*j*_ is the probability of membership in group *j*, and the conditional distribution of *Y*_*i*_ given membership in *j* is indexed by the unknown parameter vector *θ*_*j*_. Typically, the trajectory is modeled by a polynomial function of time (or age). For the case where ***P***(***Y***_***i***_| ***j***; ***θ***_***j***_) is assumed to follow the beta distribution, its mean at time t for group j, *μ*_*jt*_, is linked to time as follows:

$$ {\mu}_{jt}={\beta}_{0j}+{\beta}_{1j}t+{\beta}_{2j}{t}^2\dots . $$

where, in principle, the polynomial can be of any order.^1^ Note that the parameters linking *μ*_*jt*_ to time are trajectory group specific, thus allowing the shapes of trajectories to vary freely across group. Also associated with each trajectory group is a group specific precision parameter, *ϕ*_*j*_. The remaining components of *θ*_*j*_ pertain to the parameterization of *π*_*j*_, which in this case is specified to follow a multinomial logistic function.

For given *j*, conditional independence is assumed. In other words, except as explained by individual *i*’s trajectory group membership, serial observations in the random vector *Yi* are assumed to be independent of one another. Thus, we may write:

2 $$ {P}_k\left({Y}_i|j;{\beta}_j\right)=\prod \limits_{t=1}^T{p}_k\left({y}_{it},j;{\beta}_j\right) $$

While conditional independence is assumed at the level of the latent trajectory group, at the population level outcomes are not conditionally independent because they depend on a latent construct, trajectory group membership. See chapter 2 of [1] for a discussion of the conditional independence assumption.

The GBTM modeling framework does not require that the random vector *Y*_*i*_ be complete for all individuals. For the baseline GBTM specified above, missing values in *Y*_*i*_ are assumed missing at random. However, for applications such as that described below where measurement ends due to some external event—in this case due to the death of the patient or the patient awakening from coma—an extension of GBTM described in [13] may be used to account for non-random dropout.

Detailed discussion of the methods to approach selection of J, the number of latent groups in the population, and the order of the polynomial specifying each group’s trajectory are beyond the scope of this paper and have been previously described [1]. Briefly, no test statistics identifies the number of components in a finite mixture [14, 15]. Also, as argued in [1], in most application domains of GBTM the population is not literally composed of a finite mixture of groups. Instead the finite mixture is intended to approximate an underlying unknown continuous distribution of trajectories for the purpose of identifying and summarizing its salient features. As described in [14, 16], finite mixture models are a valuable tool for approximating an unknown continuous distribution. In this paradigm, model selection is performed by combining test statistics such as AIC and BIC, which can guide the statistician to identify which model best fits the data. This is combined with expert knowledge of which model best reveals distinctive trajectory groups that are substantively interesting. The order of the polynomial used to model each group’s trajectory is typically determined by starting with an assumed maximum order for each trajectory group then successively reducing the order if the highest order term is statistically insignificant.

All models are estimated with software that is freely available at https://www.andrew.cmu.edu/user/bjones/. The maximization is performed using a general quasi-Newton procedure [17, 18] and the variance-covariance of parameter estimates are estimated by the inverse of the information matrix.

## Results

We demonstrate use of the beta distribution in a GBTM of data quantifying brain activity of 396 comatose patients resuscitated from cardiac arrest. The University of Pittsburgh Institutional Review Board approved all aspects of this study. The data result from an observational cohort study of consecutive comatose patients hospitalized at a single academic center from April 2010 to October 2014 that underwent continuous electroencephalographic (EEG) monitoring for at least 6 h after resuscitation from cardiac arrest. Not included are patients that arrested from trauma or catastrophic neurological event, and those who awakened, died or were transitioned to comfort care within 6 h of hospital arrival.

The point of departure for our demonstration is prior work that applied GBTM to an indicator of brain activity, suppression ratio, a quantitative measure of the proportion of a given EEG epoch that is suppressed below a particular voltage threshold for activity [19]. In the first hours after cardiac arrest, many patients’ EEGs are quite suppressed (50–80%) [19] showed that patients with persistently low or rapidly improving suppression ratios often make good recoveries, while persistent suppression over the first 36 h is ominous.

**Fig. 1:**
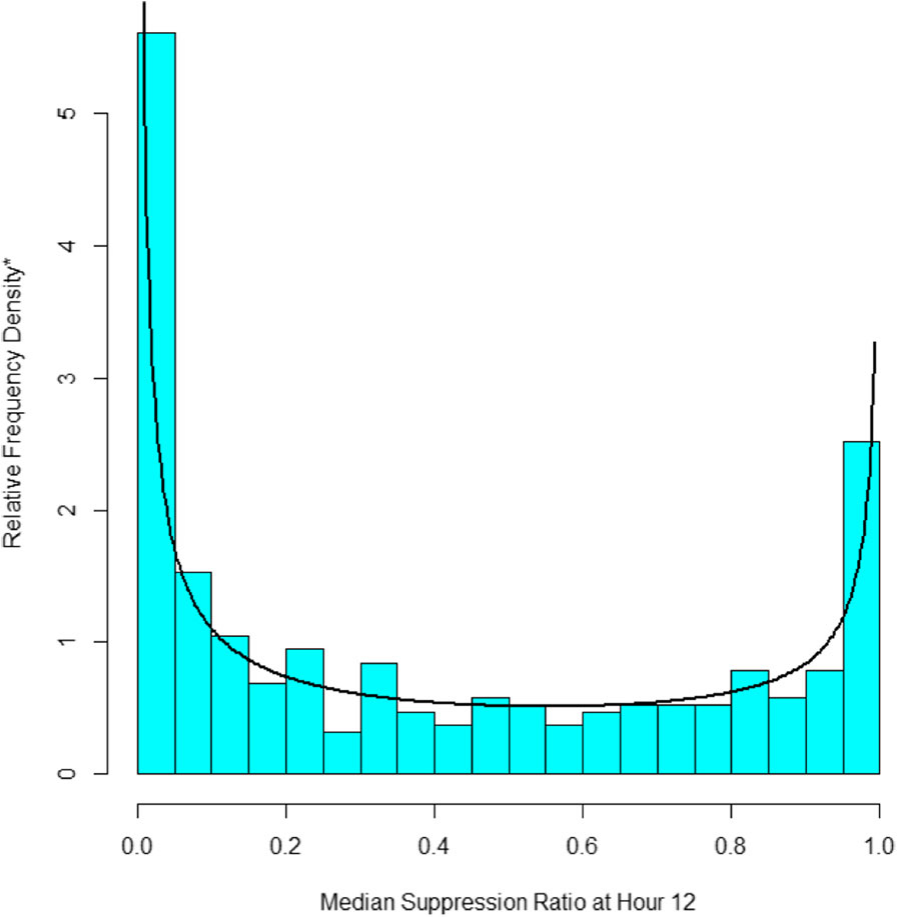
The Distribution of Hour 12 Suppression Ratio Data with the Best Fitting Beta Distribution. *The sum of the heights of the relative frequency density bars multiplied by their width sum to 1.0 so as to conform the with estimated beta density

Our main concern with the prior application was the assumption that suppression ratio followed a censored normal distribution with a minimum of 0 and a maximum of 1. To illustrate the basis for our concern, consider Fig. 1, which reports a histogram of the median suppression ratio at hour 12. It has two spikes close to the minimum of 0 and the maximum of 1. In between, the suppression ratio is approximately uniformly distributed. The histogram bears no resemblance to the normal distribution. While it is possible for a mixture of censored normal distributions to approximate the histogram in Fig. 1, the distribution of suppression ratio data within the four groups reported in [19] does not resemble the normal distribution. By contrast, overlying the histogram is a beta distribution with μ = 0.42 and ϕ = 0.77, which closely resembles the observed distribution of the suppression ratio.

**Fig. 2:**
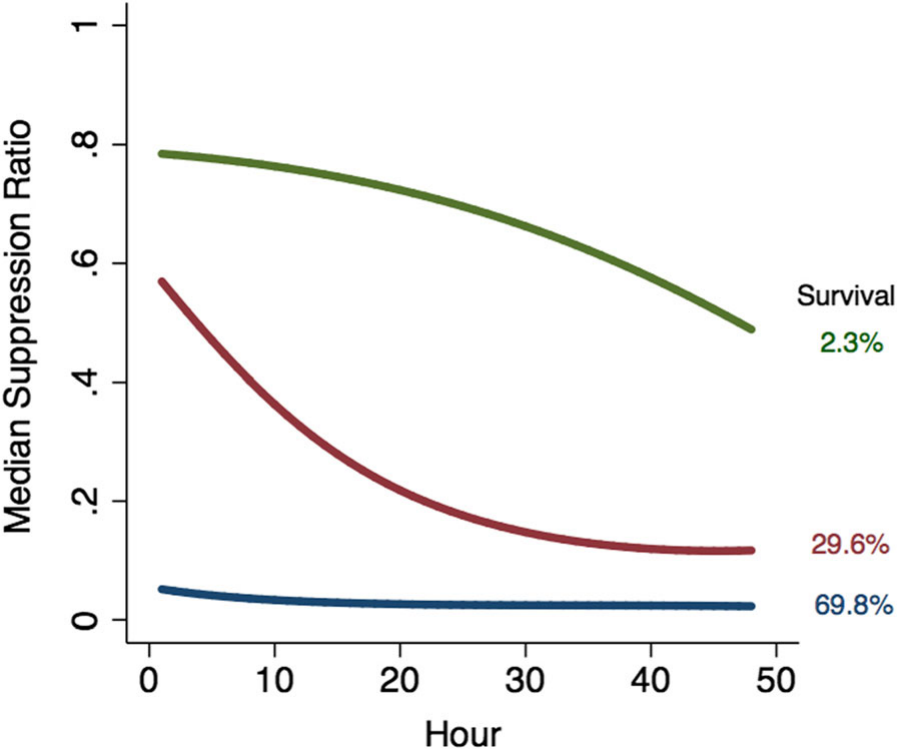
Three Group Trajectory Model with Beta Distributed Suppression Ratio

Figure 2 shows a three group, beta-based trajectory model over the first 48 h of suppression ratio measurements.^2^ Because EEG monitoring may be ended either because the patient dies or awakens, the model accounted for non-random subject attrition as described in [13]. The three group model was selected because it optimized both BIC and AIC compared to fewer groups, and models with four or more groups were sometimes unstable and did not identity additional trajectory groups that were clinically interesting in terms of their survival prospects. For the three group model, group 1 is specified to follow a cubic function of time, and groups 2 and 3 are specified to follow quadratic functions of time because as discuss above the cubic term of these trajectories were statistically insignificant at the .05 level**.** As was found in the prior analysis based on the censored normal assumption, trajectory group is strongly associated with survival probability. Overall, only about a third of patients survive to hospital discharge. However, survival probability for group 3, which accounts for an estimated 32.0% of patients who have a persistently high suppression ratio, only an estimated 2.3% survive. By contrast group 1, which accounts for an estimated 26.8% of patients, follows a persistently low suppression ratio trajectory. For this group survival probability is an estimated 69.8%. In between are group 2 patients.

**Fig. 3:**
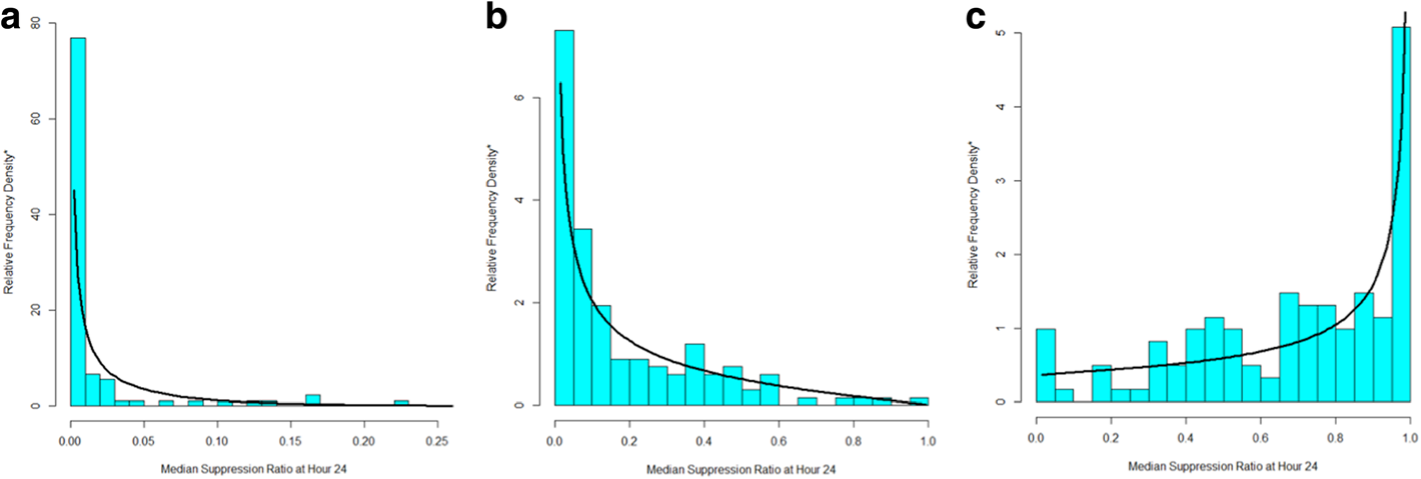
Distribution of 24 h suppression ratio data with the best-fitting data distribution for Group 1 (**a**), Group 2 (**b**) and Group 3 (**c**). *The sum of the heights of the relative frequency density bars multiplied by their width sum to 1.0 so as to conform the with estimated beta density

How well do these beta distribution-based trajectories fit the data? Fig. 3 overlays the actual distribution of the suppression ratio data by trajectory group with the predicted distribution according to the beta distribution at hour 24. Inspection of the Figure reveals that for each trajectory group the actual and predicted values nicely correspond even though across trajectory group the distribution of the suppression ratio are quite different. Trajectory group 1 (Fig. 3a) and trajectory group 2 (Fig. 3b) have right skewed suppression ratio distributions, whereas the distribution for trajectory group 3 (Fig. 3c) is left skewed. Moreover, the left skew of groups 1 and 2 are distinctly different, with group 1’s skew far more extreme than group 2’s. The fit between the actual and predicted data distribution by trajectory group is similarly good for other hours.

## Discussion

We note that the use of the beta distribution does require an adjustment for boundary observations, namely data equal to 0 or 1, which are formally not feasible for a beta distributed random variable. For boundary observations we follow the suggestion of [20] and add/subtract from 0/1 data points a small amount equal to .5 divided by the number of subjects, 396. However, a useful generalization to avoid this ad hoc adjustment would be the addition of the equivalent of the zero-inflation factor in the Poisson distribution to account for data at the boundary values of the beta distribution.

## Conclusion

We have demonstrated an extension of GBTM that adds the beta distribution to the heretofore usually applied distributions for modeling trajectories—the censored normal, zero-inflated Poisson, and binary logit. The beta option provides an alternative to the censored normal distribution for modeling continuous or approximately continuous measured outcomes measured over age or time. Figure 1 makes clear that the normal distribution poorly fits the suppression ratio data whereas the beta distribution provides a far better fit. Figure 3 also makes clear that due to the flexibility of the beta distribution a beta-based GBTM can accommodate differences in the distribution of the suppression ratio across trajectory group and over time that are not readily accommodated by the normal distribution.

## Abbreviations

EEG: Electroencephalography
GBTM: Group-based trajectory modeling
ML: Machine learning
PPGM: Posterior probability of group membership
SR: Suppression ratio

## Footnotes

1 Up to 5th order polynomials can be estimated in the software used to estimate the models reported in the case study.

2 The call to the Stata-based trajectory estimation used to estimate this model was as follows: traj, var.(srt1-srt48) indep(t1-t48) model(beta) order(3 2 2) dropout(0 0 0) where srt* is the median supression ratio at hour * and t* is the hour of measurement from 1 to 48 and the “dropout” component of the call activates the generalization of GBTM to account for nonrandom subject attrition.
